# Research Progress on Color Image Quality Assessment

**DOI:** 10.3390/jimaging11090307

**Published:** 2025-09-08

**Authors:** Minjuan Gao, Chenye Song, Qiaorong Zhang, Xuande Zhang, Yankang Li, Fujiang Yuan

**Affiliations:** 1 School of Computer Science and Technology, Taiyuan Normal University, Jinzhong 030619, China; songchenye7037@link.tyut.edu.cn (C.S.); m202375390@hust.edu.cn (Q.Z.); 202325502014@stu.tynu.edu.cn (Y.L.); yuanfujiang@ctbu.edu.cn (F.Y.); 2 School of Electronic Information and Artificial Intelligence, Shaanxi University of Science and Technology, Xi’an 710021, China; zhangxuande@sust.edu.cn

**Keywords:** color image quality assessment, full-reference methods, no-reference methods, human visual system

## Abstract

Image quality assessment (IQA) aims to measure the consistency between an objective algorithm output and a subjective perception measurement. This article focuses on this complex relationship in the context of color image scenarios—color image quality assessment (CIQA). This review systematically investigates CIQA applications in image compression, processing optimization, and domain-specific scenarios, analyzes benchmark datasets and assessment metrics, and categorizes CIQA algorithms into full-reference (FR), reduced-reference (RR) and no-reference (NR) methods. In this study, color images are evaluated using a newly developed CIQA framework. Focusing on FR and NR methods, FR methods leverage reference images with machine learning, visual perception models, and mathematical frameworks, while NR methods utilize distortion-only features through feature fusion and extraction techniques. Specialized CIQA algorithms are developed for robotics, low-light, and underwater imaging. Despite progress, challenges remain in cross-domain adaptability, generalization, and contextualized assessment. Future directions may include prototype-based cross-domain adaptation, fidelity–structure balancing, spatiotemporal consistency integration, and CIQA–restoration synergy to meet emerging demands.

## 1. Introduction

**Figure 1:**
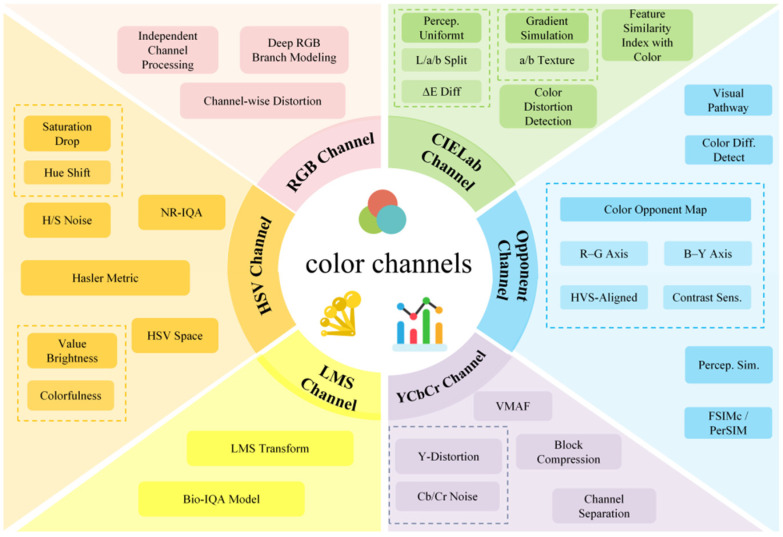
Types of color image channels. Each colored sector corresponds to a different color space (RGB, HSV, CIELab, Opponent, LMS, YCbCr). Solid boxes represent representative methods, models, or distortion types, whereas dotted boxes indicate perceptual attributes, feature dimensions, or evaluation metrics. The central icons illustrate the overall categorization framework of color channels in CIQA.

With the rapid development of computer vision, deep learning, and multimedia technology, image processing technology has been widely used in various fields, including medical image analysis [1], intelligent remote sensing [2], autonomous driving [3,4], virtual reality [5], etc. In the early stages of research, grayscale images were widely used in image analysis and processing due to their simplicity and low computational cost. However, as research has progressed, color images have garnered increasing attention due to their richer visual information, including detail, object recognition, spatial perception, and emotional expression, and have therefore become more prominent in modern image processing applications. Unlike grayscale images, which contain only luminance and luminance contrast information, color image representations encode multiple perceptual dimensions. For example, the luminance–chromaticity space (YCbCr) separates brightness from color components; the HSV space describes color using hue (H), saturation (S), and value (V); and perceptually uniform spaces such as CIELAB represent color using L (lightness), a (red–green axis), and b (yellow–blue axis), as shown in Figure 1. These dimensions have varying degrees of sensitivity to color visual quality (CVQ): slight luminance and luminance contrast noise may not alter hue, while chroma shift or saturation compression can significantly impact subjective quality and inter-channel correlation. Therefore, IQA methods that rely solely on grayscale statistics and do not explicitly model cross-channel dependencies or color perception typically fail to capture distortions such as color aberration, color differences, and cross-channel masking effects.

Although there have been a large number of reviews on existing image quality assessment methods, most of them focus on grayscale images, and there is a lack of special discussion on the quality assessment of color images. Traditional IQA methods are mostly based on the statistical characteristics of grayscale images. The damage to the quality of color images is not only reflected in the change in the brightness component [6], but also involves the distortion of the chrominance component [7]. These factors have a significant impact on the audience’s visual perception. Therefore, existing grayscale-oriented IQA methods may be inadequate for handling multidimensional color information, particularly in assessing chromatic distortions, color difference variations, and perceptual consistency. IQA methods can be divided into three categories: full-reference (FR), reduced-reference (RR), and no-reference (NR) [8]. FR requires a distorted image and a complete reference image, RR only requires a distorted image and a partial reference image, and NR only uses a distorted image. The application effect of traditional grayscale IQA methods in color images is not ideal. Structural similarity (SSIM) [9], Information Fidelity Criterion (IFC) [10], Visual Signal-to-Noise Ratio (VSNR) [11], Feature SIMilarity (FSIM) [12], and other algorithms have improved the accuracy of image assessment, but there is scope for further development. Moreover, most of them only target the grayscale information of the image, so it is of great significance to study the review of CIQA.

Reviewing the research progress of CIQA can help the academic community identify the limitations of existing methods, clarify future research priorities, and provide theoretical guidance for new algorithms, thereby improving the accuracy and wide applicability of CIQA.

## 2. Methodology

### 2.1. Search Databases and Time Range

This paper systematically searched databases including Web of Science, Scopus, IEEE Xplore, ACM Digital Library, and arXiv, with the search period set from 2000 to 2025.

### 2.2. Search Keywords and Logical Formulas

The search formula was constructed using a Boolean combination:

(“image quality assessment” or IQA or CIQA) AND (color or chromaticity or color difference or CIELAB or HSV) AND (full-reference or reduced-reference or no-reference or FR or RR or NR or dataset or database or metric)

The search formula was also expanded to include typical database and metric names, such as LIVE, CSIQ, TID2013, KADID-10k, KonIQ-10k, CCID2014, CEED2016, UIQD, PLCC, SROCC, RMSE, MAE, Kendall’s tau, etc.

### 2.3. Inclusion Criteria

(i) Focus on color image quality;
(ii) Include subjective quality experiments or objective metrics related to color perception;
(iii) Primarily peer-reviewed journals or mainstream conference papers, supplemented by high-impact preprints where necessary;
(iv) Cover full-reference (FR), reduced-reference (RR), and no-reference (NR) methods, with a focus on their application and evaluation in color image scenarios.

### 2.4. Screening and Duplicate Removal Process

Candidate papers were first screened based on title and abstract, followed by a full-text review. Duplicates were removed by merging titles, DOIs, and metadata. The screening process was performed independently by two authors, with a third author mediating any disagreements.

### 2.5. Data Extraction and Quality Control

To ensure consistency, we uniformly extracted the following information from included articles:

(i) Method category, whether explicit color or cross-channel modeling was included;
(ii) Training/test datasets used and subjective experimental design;
(iii) Evaluation metrics and statistical methods employed.

### 2.6. Number of Literature and Statistical Results

During the initial search phase, a total of 468 candidate articles were obtained. After title and abstract screening, 253 articles were retained. After full-text review and deduplication, 125 core articles were finally included. This ensured the systematicity and comprehensiveness of the review.

### 2.7. Research Framework

**Figure 2:**
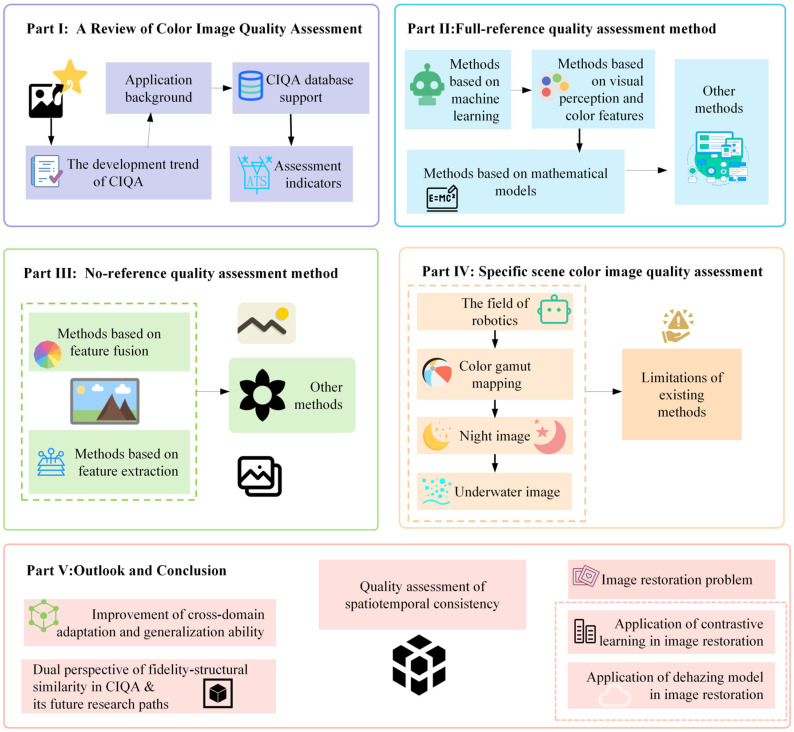
The main overview diagram of this article. Different colors indicate the five main parts of this review; icons represent specific categories or concepts; arrows denote the flow of information; dotted boxes highlight subcategories or additional methods.

This paper first reviewed the current application status of CIQA, systematically analyzed relevant databases and evaluation metrics, and focused on the current mainstream full-reference and no-reference image quality assessment algorithms. Addressing the limitations of existing technologies, this paper proposes several potential future research directions, specifically focusing on improving cross-domain adaptation and generalization capabilities, balancing fidelity and structural similarity in CIQA, building a spatiotemporal consistency quality assessment system, and addressing related issues in image restoration. To more intuitively present the research framework and technical context of this paper, the main overview diagram of this paper is shown in Figure 2.

## 3. A Review of Color Image Quality Assessment

Before we delve into the specific methods of CIQA, we first need to understand its application background, the database it relies on, and the selection of assessment indicators. The following discussion will be carried out addressing three aspects, the application status of CIQA, related databases, and assessment indicators, to further provide theoretical support and practical basis for subsequent research.

**Figure 3:**
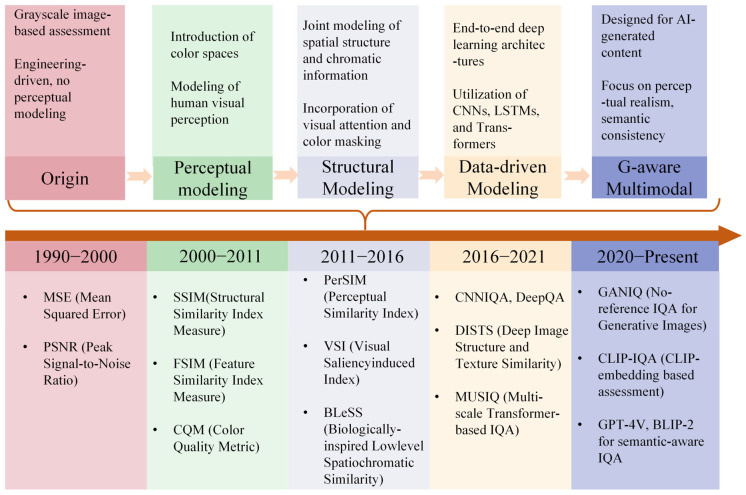
The development trend of CIQA.

Building on this foundation, it is important to examine the evolution of CIQA technologies over time. As illustrated in Figure 3, the development of CIQA can be divided into five stages, each reflecting advances in technology and perceptual modeling. In the origin stage (1990–2000), image quality assessment was mainly based on traditional signal error metrics, such as mean squared error (MSE) and peak signal-to-noise ratio (PSNR). Although these methods were not originally designed for images, their widespread application began in image compression and transmission assessment practices, representing the earliest “engineering-oriented” quality assessment ideas. These methods focus on engineering implementation and ignore the characteristics of human visual perception. Entering the perceptual modeling stage (2000–2011), researchers began to introduce color spaces such as Lab and YUV, and tried to simulate the visual mechanism of the human eye. Representative methods include SSIM [9], FSIM [12], and CQM [13]. Subsequently, in the structural modeling stage (2011–2016), the methods further integrated image structure and color features, emphasizing the modeling of visual attention and color masking, such as PerSIM [14], VSI [15], and BLeSS [16]. With the rise of deep learning technology, the data-driven stage (2016–2021) adopted CNN, LSTM, Transformer, and other architectures for end-to-end training. Representative methods include CNNIQA [17], DeepQA [18], DISTS [19], and MUSIQ [20]. Entering the generative adaptation and multimodal stage (2020–present), the CIQA method began to focus on AI synthetic images (such as GAN or diffusion model generated images), while integrating semantic consistency and visual realism assessment. Representative works include GIQA [21], CLIP-IQA [22], and cutting-edge exploration of quality assessment combined with multimodal large models such as GPT-4V [23] and BLIP-2 [24]. This development path reflects the leap from traditional engineering indicators to intelligent semantic perception.

### 3.1. Current Application of Color Image Quality Assessment

**Figure 4:**
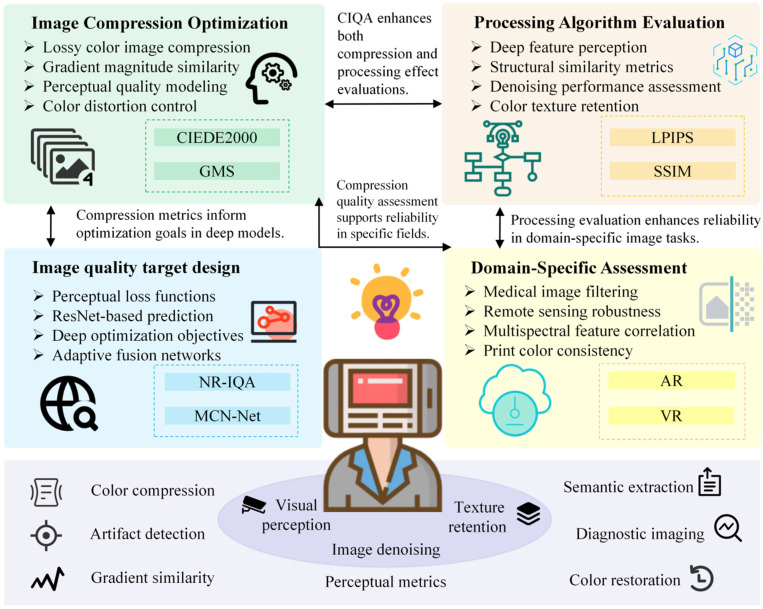
Application status of color image quality assessment. Different colors indicate four main modules (Image Compression Optimization, Processing Algorithm Evaluation, Image Quality Target Design, Domain-Specific Assessment) and the perceptual metrics—related part; icons represent specific concepts or categories; arrows denote the flow of information or influence between modules; dotted boxes highlight subcategories or key methods.

CIQA plays a key role in many practical applications. With the advancement of image processing technology, accurate quality assessment has become the basis for optimizing processing algorithms and compression standards. CIQA methods are constantly evolving to meet the needs of image quality in different fields. Next, we will briefly review the current application status of CIQA in image compression, processing optimization, target design, and specific fields. Figure 4 shows the current application status of CIQA. In Section 2.3 of this article, we discuss in detail the current research progress in specific research fields.

(i) Used for image compression and coding optimization

Color image compression is an inevitable part of information transmission and storage. In particular, in lossy compression, image quality is often affected [25]. For color images, it is necessary not only to ensure the clarity of brightness information, but also to maintain color details and natural transitions as much as possible to avoid color distortion. The Reduced-Reference Video Quality Assessment (RR VQA) method can be transferred to the image compression field by combining external ventricular drainage (EVD) and generalized Gaussian density (GGD) [26], and is used for compression quality monitoring, algorithm optimization, artifact detection and repair tasks. The gradient magnitude similarity (GMS) [27] in the full-reference method provides a new technical path for CIQA by quantifying the consistency of the gradient structure between the reference image and the distorted image. Quality assessment methods based on perceptual models, such as the CIEDE2000 color difference model, have been introduced into the image compression field [28] to more accurately evaluate the perceptual quality of color images. These assessment methods can guide the encoder to balance the compression ratio and visual quality in image compression coding. For color images, the improvement of the assessment criteria has a more significant impact on the final image quality.

(ii) Performance assessment of image processing and enhancement algorithms

In practical applications, image processing and enhancement algorithms [29] are often optimized for specific tasks, such as image denoising [30], super-resolution reconstruction [31], and image deblurring [32]. It is important to note that these are target algorithms whose output quality needs to be evaluated, rather than assessment methods themselves. For color images, traditional assessment indicators, such as mean squared error (MSE), often fail to reflect subtle color changes, resulting in a distorted assessment of the effect after image processing. SSIM and multiscale SSIM (MS-SSIM) [33] are widely used in IQA. They can more accurately reflect the perceptual quality of images by quantifying the SSIM between images, and are widely used in image processing, denoising, and other tasks. However, with the development of deep learning technology, perceptual assessment indicators based on deep features have gradually become a new choice.

Learned Perceptual Image Patch Similarity [34] (LPIPS) can better simulate human visual perception [35] by extracting image features using deep neural networks, and performs well in the performance assessment of image processing and enhancement algorithms. Compared with traditional SSIM and MS-SSIM, LPIPS can not only capture the structural information of the image, but also more accurately reflect perceptual characteristics such as color and texture, thereby more comprehensively evaluating the effect of the image processing algorithm. For example, in the field of image denoising, using LPIPS to evaluate image quality can not only retain the main structural information of color images, but also better reduce the loss of color details in the denoising process, thereby improving the subjective visual effect of color images. Thus, the performance of these enhancement algorithms can be evaluated using CIQA methods, which provide objective measures to assess the perceptual quality of their output, demonstrating the utility of assessment metrics in image processing research.

(iii) Design of image quality optimization targets

With the rapid development of deep learning technology, IQA-based target optimization has gradually become an important direction in the design of image processing algorithms. In tasks such as image restoration, super-resolution reconstruction, and compression design, traditional optimization methods based on error metrics may not be able to fully capture the complex quality features of color images in some cases. Therefore, by introducing non-convex optimization theory [36], quality assessment indicators that are more in line with human visual perception characteristics have gradually become a research trend in the field of image processing.

In neural network-based image processing, the NR-IQA method MCN-Net combines ResNet-50 with a perception module to extract multi-scale semantic features, and uses an adaptive fusion network to optimize them, enabling high-quality image prediction [37]. With the continuous advancement of deep neural network technology, deep learning models based on perception optimization are expected to become the enabling technologies in the field of color image processing in the future. For example, in areas such as image processing in smartphones, image analysis in autonomous driving, and content generation for virtual reality (VR) and augmented reality (AR), higher image quality can be achieved through perceptual optimization, thereby improving user experience.

(iv) Quality assessment for specific fields

CIQA plays a vital role in many specific fields. In the field of medical imaging, the quality of color images directly affects the diagnosis and treatment decisions of diseases [38]. Medical images are often limited by the performance of the acquisition equipment and the noise and blur that may occur during the scanning process. Automated quality assessment tools can effectively improve the diagnostic efficiency of medical images. In this field, IQA can not only automatically screen out images with unqualified quality, but can also provide a basis for subsequent image processing and enhancement. In the field of remote sensing images, the accuracy of IQA directly affects the accuracy of surface monitoring and the reliability of meteorological forecasts. Remote sensing images are often affected by factors such as equipment performance and atmospheric interference, and are prone to noise, blur, and other problems [39]. The introduction of an adaptive band selection strategy driven by an attention mechanism [40] can dynamically capture the global correlation of multispectral features. At the same time, IQA can automatically screen out low-quality images, ensure data reliability, and provide guidance for image denoising, enhancement, and restoration. In the field of printing, accurate color restoration is a key issue. Texture feature analysis based on active contours [41], combined with adaptive attention weight allocation, can optimize the robustness of color difference detection. By using the CIQA method, the color difference between the printed image and the original image can be objectively evaluated to ensure color consistency and accuracy.

### 3.2. Color Image Database

The existing image quality databases mainly include LIVE [42], CSIQ [43], TID2013 [44], KADID-10k [45], KonIQ-10k [46], QACS [47], CCID2014 [48], CEED2016 [49], 4K Resolution Enhancement Artifact Database [50], UIQD [51], AGIQA-1k [52], AGIQA-3K [53], AIGCIQA2023 [54], PKU-I2IQA [55], etc. Table 1 lists the relevant parameters of nine representative color image quality assessment datasets from 2006 to 2023. Here, we introduce five commonly used color image quality databases. These databases have promoted the research of CIQA from different dimensions and have important theoretical and practical significance.

(i) Quality Assessment of Compressed SCIs (QACS)

The QACS database was built by Nanyang Technological University, Singapore, in 2016 to evaluate the quality of compressed color SCIs. The database contains 492 distorted images from 24 high-quality reference SCIs. These distorted images are generated by two compression technologies, High Efficiency Video Coding (HEVC) and Screen Content Coding (HEVC-SCC). Each technology is further divided into 11 different distortion levels, which are distinguished by the quantization parameter QP value. The subjective experiment follows the principle of the single stimulus method, and invites subjects to score the image quality. The score is presented in the form of mean opinion score (MOS), which is defined between [1,10]. The higher the score, the better the image quality. The QACS database aims to provide a standardized reference benchmark for the quality assessment of compressed SCIs.

(ii) Contrast-Changed Image Database (CCID2014)

The CCID2014 dataset was developed by Shanghai Jiao Tong University in 2016 to study the impact of contrast changes on color image quality. The database comprehensively covers 15 high-quality original color images, and generates 655 contrast-adjusted images (EIs) based on them. Subjective scores were provided by 22 subjects, ranging from [1,5], where lower scores indicate worse image quality. These scores were obtained through subjective experiments and aim to provide a standardized reference for IQA.

(iii) Contrast enhancement assessment database (CEED2016)

CEED2016 was established by Islamia University, Bahawalpur, Pakistan, in 2017 [49]. It is a color image database focused on contrast enhancement assessment. It contains 30 original color images and 180 EIs generated by six different contrast image enhancement (IE) algorithms. A total of 23 subjects participated in the subjective experiment to obtain the subjective quality score of EI.

(iv) 4K Resolution Enhancement Artifact Database

This database was constructed by Zheng et al. in 2022 [50]. The database includes 24 original 4K images, 480 degraded low-resolution images, and 1152 resolution-enhanced 4K images. The quality score of the database is subjectively scored by more than 20,000 observers. This database provides valuable data support for the research of super-resolution technology, and is particularly helpful in optimizing the existing CIQA method.

(v) Underwater Image Quality Database (UIQD)

The UIQD database is a large-scale underwater image quality assessment database released by Liu et al. in 2024 [51]. The database contains 5369 real underwater images, covering a variety of underwater scenes and typical quality degradation conditions such as blur, noise, color distortion, etc.

### 3.3. Assessment Metrics

#### 3.3.1. Classification of Metrics

For ease of comparison and interpretation, this paper categorizes commonly used image quality assessment metrics into two categories:

(i) Correlation-based metrics, which measure the statistical association between objective prediction scores and subjective scores. Common metrics include the Pearson correlation coefficient r (PLCC), the Spearman rank correlation coefficient ρ (SROCC), and Kendall’s tau (τ). PLCC primarily assesses linear consistency, while SROCC and Kendall’s tau measure the strength and direction of monotonic relationships using ranked methods, making them well suited for non-continuous or ordinal variables. In particular, SROCC evaluates the consistency of relative rankings, whereas Kendall’s tau further assesses pairwise ranking agreements, providing complementary perspectives on monotonic associations.
(ii) Error-based metrics, which quantify the numerical deviation between the predicted score and the subjective true value. Common metrics include mean absolute error (MAE) and root mean square error (RMSE), but these metrics depend on the database dimension.

**Figure 5:**
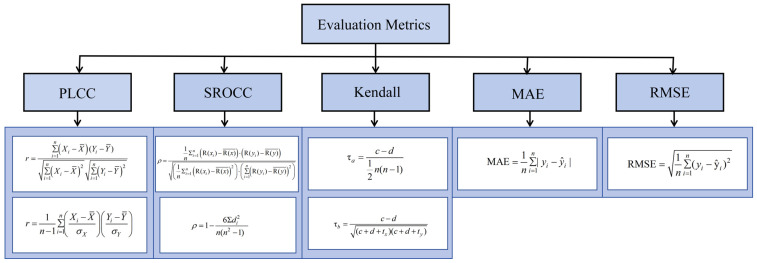
Formula diagram.

Specifically, higher PLCC and SROCC values and lower MAE and RMSE values indicate higher prediction accuracy, monotonicity, and consistency. Ideally, PLCC = 1, SROCC = 1, RMSE = 0, and MAE = 0, indicating perfect agreement between the objective and subjective evaluations. The corresponding mathematical expressions are shown in Figure 5.

#### 3.3.2. Experimental Comparison Across Databases

To further illustrate the differences between these metrics, we conducted an experiment using peak signal-to-noise ratio (PSNR) as the baseline quality metric, as shown in Table 2. The performance of PSNR is evaluated on three widely used IQA databases: LIVE, CSIQ, and TID2013. Basic information about these three databases is provided in Table 1. Overview of the Data set in Section 3.2.

#### 3.3.3. Metric Analysis and Advantages and Disadvantages

To fully understand the image quality assessment results, it is necessary to conduct an in-depth discussion of various metrics, combining theoretical characteristics with experimental performance. Correlation metrics (PLCC, SROCC, Kendall’s τ) primarily reflect the degree of consistency between the predicted results and subjective opinions, while error metrics (MAE, RMSE) characterize prediction accuracy from the perspective of numerical deviation. The advantages and disadvantages of different metrics directly determine their applicability in practical applications.

(i) PLCC

The advantage of PLCC is that it directly measures the linear consistency between the predicted results and the subjective scores after performing nonlinear regression mapping. Its value is intuitive and interpretable. On the LIVE database, the PLCC for PSNR reached 0.8736, indicating that, after regression correction, a strong linear relationship was maintained between the predicted and subjective scores. Therefore, PLCC is highly valuable in evaluating overall fitting accuracy and the model’s ability to linearly approximate subjective perception. However, PLCC is highly sensitive to outliers and often underestimates performance when the true relationship is monotonic but nonlinear, a phenomenon evident in the TID2013 database. Furthermore, the size of PLCC is affected by database distribution and dimensionality differences, making it prone to bias and misinterpretation if used directly for cross-database comparisons.

(ii) SROCC

The advantage of SROCC lies in its reliance solely on rank information. By first regressing the predicted values and then calculating the rank correlation, it ensures that it remains invariant to any monotonic transformation and is therefore unaffected by dimensionality, providing a more stable reflection of ranking consistency. The theoretical basis of SROCC lies in its ability to measure monotonic associations rather than strict linearity, making it particularly effective when subjective scores are discrete, ordinal, or non-continuous. In this experiment, the SROCC values of PSNR on LIVE and CSIQ were 0.8781 and 0.8052, respectively, demonstrating cross-database stability.

However, SROCC cannot reflect the absolute error between the predicted values and the subjective scores, and can easily overlook systematic bias. In databases like TID2013, where the rating scale is relatively discrete and there are a large number of tied ranks, the discriminative power of SROCC is somewhat affected, resulting in a decrease in the value.

(iii) Kendall’s τ

The advantage of Kendall’s τ is that it measures rank consistency based on pairwise sample comparisons and is robust to tied rankings. Therefore, it maintains good reliability in databases with a limited range of subjective scores and a large number of identical ratings. Like SROCC, Kendall’s τ focuses on monotonic relationships and is grounded in ranked methods, but it differs by emphasizing pairwise concordance and discordance, thereby providing stronger interpretability for ordinal and non-continuous subjective ratings. In experiments, its numerical trend is generally consistent with SROCC, providing complementary verification of ranking consistency.

The disadvantage is that the computational complexity increases significantly with large sample sizes, but its statistical power is higher, allowing it to more reliably reveal the significance of ranking relationships under large sample conditions. Furthermore, because the numerical magnitude of Kendall’s τ is generally lower than SROCC, direct comparisons can easily lead to misinterpretations of performance underestimation.

(iv) MAE

The advantage of MAE is its simple and intuitive calculation method, which reflects the average deviation between the predicted and subjective scores. Compared to RMSE, it is less sensitive to outliers and has more stable results. Its value directly corresponds to the database’s dimension. For example, it is approximately 10, 0.12, and 0.98 on LIVE, CSIQ, and TID2013, respectively, providing an intuitive reference for deviation in application scenarios.

However, MAE is highly dependent on the database’s score range, making it difficult to directly compare values across databases. Furthermore, MAE lacks sufficient sensitivity to small, severe prediction errors, and may not fully reveal model instability in high-risk or mission-critical applications.

(v) RMSE

The advantage of RMSE is that it enhances sensitivity to large deviations through a squared penalty, highlighting model weaknesses under catastrophic predictions. Therefore, it is particularly important in tasks that emphasize stability and safety. In experiments, RMSE was approximately 13 on LIVE, clearly demonstrating its significant amplification of large errors.

However, RMSE is highly sensitive to outliers; even a small number of deviations can dominate the overall results, obscuring the overall trend. At the same time, RMSE, like MAE, is strongly dependent on the dimension. The values between different databases cannot be directly compared. If it is not standardized, its explanatory power will be significantly reduced.

In summary, correlation indicators can reveal the consistency between prediction results and subjective evaluations, and are more suitable for scenarios across databases or with inconsistent dimensions; error indicators can provide an intuitive depiction of numerical deviations, and are more in line with the sensitive requirements of practical applications for error ranges.

## 4. Overview of Color Image Quality Assessment Algorithms

IQA methods can be divided into FR, RR, and NR. Next, we will explore the related research of FR and NR methods and their applications in different fields.

### 4.1. Full-Reference Quality Assessment Method

**Figure 6:**
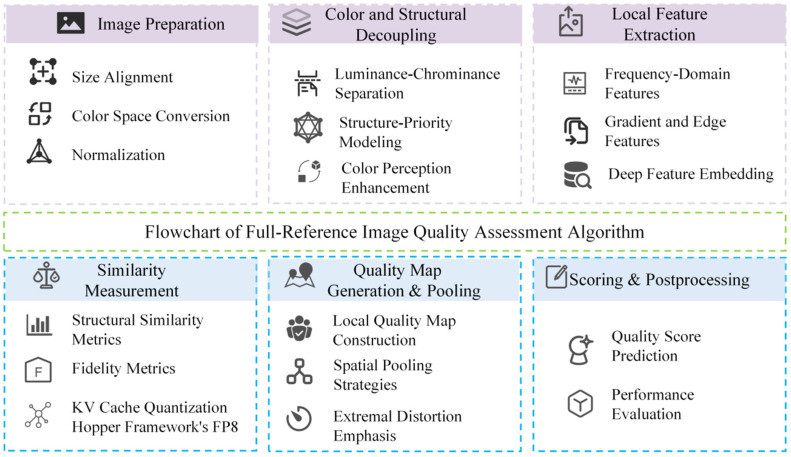
Flowchart of full-reference image quality assessment algorithm.

FR-IQA is one of the most widely studied types in the field of IQA. The FR-CIQA algorithm flow is shown in Figure 6.

#### 4.1.1. Machine Learning-Based Methods

Machine learning methods [56] are usually able to process complex image data and optimize assessment accuracy by training models.

Before 2019, Charrier et al. [57] proposed a full-reference image quality assessment algorithm based on machine learning. The algorithm first classifies images into five ITU-recommended quality levels using multi-SVM classification, and then applies a specific SVM regression model within each category for quality scoring. This method combines classification and regression to achieve a detailed assessment of image quality, effectively improving the consistency of the assessment results with human subjective assessment. Ding et al. [19] proposed Deep Image Structure and Texture Similarity (DISTS), unifying structural and texture similarity. Via CNN, it converts images into multi-scale overcomplete representations, captures texture via feature map spatial average, and measures structure with feature map spatial correlation. It shows high consistency with human perception on databases, and a robustness to geometric transformations. Also noteworthy, outside the core field of IQA, some related classification-oriented research, such as Kent et al. [58], demonstrated the feasibility of using decision tree models to predict human visual preferences. These studies indirectly suggest that supplementary metrics such as accuracy and F-score may also provide useful references for machine learning-based CIQA methods.

In 2021, Ding et al. [59] systematically compared the application of multiple FR-IQA models in optimizing image processing systems. Using eleven FR-IQA models as optimization targets, deep neural networks were trained to perform low-level visual tasks such as image denoising, deblurring, super-resolution, and compression. Through large-scale subjective tests, the perceptual performance of each model on optimized images was evaluated, revealing the relative advantages and disadvantages of different models. In 2022, Cao et al. [60] proposed a method that combines semi-supervised learning and positive-unlabeled (PU) learning. This method uses PU learning to identify abnormal samples in unlabeled data and uses semi-supervised learning to dynamically generate pseudo-MOS values. At the same time, a dual-branch network structure is used, combined with spatial attention and local slice Wasserstein distance to enhance attention to image information areas and solve alignment problems. Although the training stage leverages semi-supervised and PU strategies to overcome the shortage of labeled data, the model remains a full-reference IQA approach because, during inference, it requires both the reference and distorted images to compute the quality score. Experiments show that this method performs well on multiple benchmark datasets. In 2023, Lang et al. [61] proposed an FR-IQA model based on deep meta-learning and Conformer, combining Conformer with a twin network to extract feature vectors of reference and distorted images and calculating similarity as a prediction score. Meta-learning is used to identify distortion types, and Conformer obtains global and local features. The generalization ability is improved through double-layer gradient descent and fine-tuning, which is competitive on three standard datasets. Reddy et al. [62] proposed a practical image quality assessment algorithm based on a machine learning model. The algorithm extracts image features through multiple feature descriptors, and uses KNN matching and inlier ratio to evaluate image quality, achieving the accurate assessment of image features. In 2025, Lan et al. [63] proposed an innovative hierarchical degradation-aware network. The network extracts multi-level and multi-dimensional feature representations by simulating the degradation process of an image from a reference image to a distorted image. The similarity matrix is used to quantify the correlation between features, and the features most relevant to the distortion are selected for fusion. Finally, the fused features are mapped to quality scores through a regression network, achieving high-precision assessment of complex distorted images and significantly improving the adaptability and robustness of the assessment model.

Table 3 is constructed based on the above content. FR-IQA methods show an evolutionary trend from traditional machine learning to deep learning, and then to fusion strategies. Machine learning-based methods achieve fine assessment by combining classification and regression; deep learning models use convolutional neural networks to capture high-order features, unify structure and texture similarity, and improve consistency with human perception. Fusion strategies combine the advantages of traditional methods and deep learning, taking into account multi-dimensional information. In addition, new technologies such as semi-supervised learning and meta-learning have also been introduced to further enhance the generalization ability and robustness of the model, promoting progress in the field of FR-IQA.

#### 4.1.2. Methods Based on Color Visual Quality and Color Characteristics

The CIQA method based on color visual quality (CVQ) and color characteristics focuses on the human perception of attributes such as color, contrast, and color distribution. Many of these methods are inspired by the human visual system (HVS) in order to better approximate CVQ. Color characteristics are the core of CIQA.

Before 2019, Thakur et al. [64] proposed a new color image quality index Q_new_ to overcome the limitations of existing methods. This method combines HVS theory with IQA technology, and integrates brightness similarity, structural correlation, edge similarity, and color similarity indicators to ensure simple calculation and applicability to diverse image processing scenarios. Niu et al. [65] proposed an IQA method for color correction. This method evaluates the color consistency between the color correction image and the reference image through color contrast similarity and color value difference, thereby improving the accuracy of the assessment.

In 2019, Kuo et al. [28] proposed an IQA algorithm that comprehensively considers grayscale and color information. The chroma channel is introduced into the S-VIF algorithm to improve the performance of the algorithm. In 2020, Alsmadi et al. [66] proposed a content-based image retrieval (CBIR) system based on color, shape, and texture. The system extracts shape features through an RGB color and neutral clustering algorithm, using the Canny edge method, color features through a YCbCr color and discrete wavelet transform and Canny edge histogram, and texture features through a grayscale co-occurrence matrix. The genetic algorithm and simulated annealing algorithm are combined for similarity assessment to achieve efficient and accurate image retrieval. Experimental results show that the system outperforms other advanced CBIR systems in terms of precision and recall. In 2021, Cheon et al. [67] proposed Image Quality Transformer (IQT), the first to apply Transformer encoder–decoder to FR-IQA. It extracts deep perceptual features of reference and distorted images via CNN backbone (Inception-ResNet-v2), and constructs feature differential representation as encoder input. The Transformer layer introduces learnable quality/position embedding and uses multi-head attention to model distortion globally; the decoder takes reference features as queries to interact with encoder output; and the MLP head regresses quality scores. Shi et al. [68] proposed an FR-IQA method based on three-feature three-step fusion, namely, features fusion similarity index (FFS). This method achieves efficient comparison between the reference image and the distorted image by fusing the brightness channel, similarity map, and multi-dimensional features, and adopts a symmetric calculation method. After multi-step fusion and bias pooling strategy, the final quality score is derived. Experiments show that the FFS method shows high consistency with subjective scores on multiple large databases and has significant computational efficiency.

Table 4 is constructed based on the above content. CIQA methods based on visual perception and color characteristics are constantly evolving, from the early Q_new_ algorithm that integrates HVS theory and multi-dimensional similarity indicators, to the method that uses color contrast and value difference for assessment, to the S-VIF algorithm that comprehensively considers grayscale and color information, all of which reflect the core position of color characteristics in IQA. New technologies such as Transformer architecture, complementary color wavelet transform, and statistical color distribution have been introduced into the IQA field, significantly improving the accuracy and efficiency of assessment. These cutting-edge studies have promoted the advancement of IQA technology and provided more accurate and efficient solutions for image and video quality assessment.

#### 4.1.3. Methods Based on Mathematical Models

CIQA methods based on mathematical models quantify image quality through mathematical theories and algorithms, such as sparse representation, gradient calculation, and similarity measurement. They can quantitatively analyze the differences between images and reference images and are widely used in image compression, restoration, and enhancement.

Before 2019, Li et al. [69] proposed a CIQA method based on sparse representation and reconstruction residual. This method uses an overcomplete color dictionary trained with natural color images to represent the reference image and the distorted image, and constructs two feature maps to measure the structure and color distortion of the image. Then, the reconstruction residual is calculated to measure the contrast change of the image, and brightness similarity is introduced to finally obtain the comprehensive quality score of the color image. Sun et al. [70] proposed a superpixel-based image quality assessment (SPSIM) algorithm, which calculates brightness, chromaticity, and gradient similarity based on perceptually meaningful superpixel image blocks, further adjusts these three features based on gradient region consistency, and finally uses texture complexity as a weighting function in the pooling stage to obtain a high consistency with the subjective score.

In 2020, Shi et al. [71] proposed an FR-IQA model based on visual saliency, color appearance, and gradient similarity, namely VCGS. The model constructs a comprehensive quality assessment system by combining visual saliency features, gradient similarity, and chromaticity similarity. The model derives the final quality score through the fusion and pooling strategy of feature similarity graphs. Experiments show that VCGS is highly consistent with subjective assessment on multiple public databases and has moderate computational complexity. In 2022, Sun et al. [72] proposed a low-light image enhancement algorithm based on improved multi-scale Retinex and artificial bee colony (ABC) algorithm optimization. The algorithm first obtains the illumination component of the original image through structure extraction and relative total variation, combines the multi-scale Retinex algorithm to obtain the reflection component, and then performs histogram equalization, bilateral gamma function correction, and bilateral filtering. At the same time, another copy of the image is subjected to histogram equalization and weighted guided image filtering enhancement. Finally, the ABC algorithm is used to optimize the weights to achieve image fusion, which effectively improves the quality of low-light images and reduces detail loss and noise. Varga et al. [73] proposed an FR-IQA method based on Grünwald–Letnikov derivatives, image gradients, and visual saliency. This method captures global changes in the image through Grünwald–Letnikov derivatives, quantifies local changes using image gradients, and combines visual saliency to weight image regions to simulate the perceptual characteristics of the human visual system. By fusing global and local information, this assessment method has demonstrated excellent performance on multiple public IQA databases, effectively improving the accuracy and robustness of image quality assessment. In 2024, Yang et al. [74] proposed an image quality assessment algorithm based on a sparse structure and subjective perception (IQA-SSSP). By quantifying the sparse structural similarity between the reference image and the distorted image, an efficient and objective assessment system was constructed by combining a low-complexity computing framework with large-scale data processing capabilities. The fusion of structural similarity and visual perception characteristics provides a more comprehensive technical path for image quality assessment and deepens the application value of sparse representation theory in objective assessment. In 2025, Bezerra et al. [75] proposed a method that combines Grünwald–Letnikov derivatives, image gradients, and visual saliency. The Grünwald–Letnikov derivatives are used to capture global changes in the image, the image gradients are used to quantify local changes, and the visual saliency weighting is used to highlight the sensitive areas of the human eye. By integrating global and local features and considering the perceptual characteristics of the human visual system, the model has performed well on multiple public IQA databases and significantly improved the accuracy and consistency of image quality assessment.

Table 5 is constructed based on the above content. CIQA methods based on mathematical models continue to innovate, and by integrating technologies such as sparse representation, gradient calculation, visual saliency, and derivative theory, the objectivity and perceptual consistency of CIQA are improved. From structural similarity quantification to global–local feature fusion, researchers continue to optimize algorithms to simulate the complex characteristics of the human visual system. In the future, with the further cross-integration of deep learning and mathematical theory, CIQA methods are expected to achieve higher-precision assessment in more complex distortion scenarios while reducing computational complexity, providing stronger technical support for image processing, compression, and enhancement.

#### 4.1.4. Other Methods

In addition, there are several other related research papers.

Before 2019, Temel et al. [76] proposed a novel multi-resolution IQA method. This method comprehensively considers color and structural similarity, aiming to simulate the perception mechanism of HVS more accurately. In the LAB color space, this method uses LoG features to capture image structural information, simulate the perception process of retinal ganglion cells, and calculate color similarity in the a and b channels to comprehensively evaluate the quality of color images. Liu et al. [77] constructed an image compression reference calculation model, which introduced a color distribution rule metric to measure the difference in color distribution rules between the reference image and the distorted image. First, the three key channels of bright channel, median channel, and dark channel are accurately extracted from the color image. Advanced fractal analysis technology is used to refine the characteristics of each channel with the help of fractal dimension. Finally, the fractal dimensions extracted from each channel are integrated to accurately predict the image quality.

In 2019, Temel et al. [78] proposed the Spectral Understanding of Multi-scale and Multi-channel Error Representations (SUMMER) algorithm, which improves on the limitations of traditional grayscale image spectral analysis in terms of color information and visual system characteristics. The performance of SUMMER was verified in three databases covering about 30 types of distortion, including key categories such as color artifacts. Athar et al. [79] conducted a performance assessment study on IQA algorithms, testing 43 FR algorithms, 7 fusion FR, and 14 NR methods, covering 9 subjective assessment datasets. In 2023, Popovic et al. [80] studied the impact of saturation and hue changes on image quality in scene perception and clarified the core concepts of subjective image quality assessment. A database containing reference images and images with modified saturation and hue was created, and the settings of the subjective tests were detailed. Through a comprehensive analysis of subjective and objective assessments, the Kendall and Spearman correlation coefficients were calculated to verify the effectiveness of the method. In 2025, Watanabe et al. [81] proposed a full-reference point cloud quality assessment method FR-MLLM based on multimodal large language models (MLLMs). They introduced three MLLM-based assessment indicators and combined them with traditional indicators to improve the assessment accuracy through support vector regression. The ability of MLLM to understand high-level visual content significantly improved the assessment performance.

Table 6 is constructed based on the above content. In general, the existing CIQA methods cover statistical feature combination [76,78], fractal analysis [77], and large-scale assessment [79,80]. The latest research has further integrated multimodal LLM [81]. There is a trade-off between accuracy, real-time performance, adaptability and generalization ability among various methods.

### 4.2. Reduced-Reference Quality Assessment Method

RR-IQA’s basic idea is that, in evaluation, instead of a full-reference image, only a small number of reference features are transmitted or stored to compare with corresponding features from the distorted image, thereby predicting the image’s perceptual quality. Compared with full-reference methods, RR methods significantly reduce bandwidth and storage overhead; compared with no-reference methods, they also improve stability and accuracy via partial reference information. Yet recent RR-IQA research is relatively less prevalent, partly due to more specific application scenarios than FR- and NR-IQA and deep learning’s rapid progress attracting greater attention to end-to-end NR approaches.

In 2011, Soundararajan et al. [82] proposed a simplified reference image quality assessment (SSIM-RR-IQA) framework based on structural similarity estimation, leveraging the multi-scale multidirectional division regularized transform (DNT) for feature extraction. This method innovatively combines SSIM theory with simplified reference constraints, mapping the perceptual consistency between reference and distorted images via DNT-derived statistical features. By developing a distortion metric similar to SSIM, the method enables efficient quality assessment with minimal reference data. Experimental validation on six benchmark databases demonstrates a robust correlation with full-reference SSIM and human subjective judgment. Furthermore, the method explores practical applications of partial reference image restoration, such as deblurring tasks, highlighting its dual capabilities for quality assessment in visual communication systems and perceptual consistency enhancement. In 2012, Rehman et al. [83] proposed a reduced-reference entropy difference (RRED) framework for image quality assessment (IQA). This innovative approach measures distortion by using the information entropy difference between a reference image and a distorted image in the wavelet domain. By leveraging multi-scale decomposition and an error pooling strategy, this method achieves high perceptual accuracy with minimal reference data. Experimental validation on the LIVE and Tampere image databases demonstrates that this method outperforms the traditional MSE metric, achieving performance close to full-reference IQA while significantly reducing the amount of reference information. This entropy-based approach advances the development of RR-IQA by combining theoretical entropy analysis with practical perceptual evaluation, providing a novel paradigm for resource-efficient image quality assessment in visual communication systems. In 2016, Wang et al. [84] proposed a reduced-reference image quality assessment (RR-IQA) framework for screen content images (SCIs) based on visual perception modeling. This method innovatively integrates statistical feature analysis with human visual attention mechanisms, leveraging lightweight reference data to predict distortion severity. By extracting texture complexity and edge preservation metrics from distorted SCIs, the framework achieves efficient quality evaluation with minimal transmission overhead. Experimental validation across six benchmark datasets demonstrates superior performance in predicting subjective judgments compared to conventional RR-IQA models, particularly for compression artifacts and virtual desktop applications. The work, supported by multiple national research funds, explores dual-use scenarios including remote image processing and perceptual consistency optimization, marking a significant advance in practical IQA for screen content visualization systems. In 2022, Yu et al. [85] proposed a reduced-reference perceptual hashing framework perceptual hashing algorithm based on complementary color wavelet transform (CCWT) and compressed sensing (CS). The algorithm decomposes the input color image into different subbands through CCWT, retains all color channel information, and uses block-based CS to extract features from the subbands to construct perceptual features with strong robustness, discrimination, and compactness. By quantizing the perceptual features to generate hash sequences, excellent performance in IQA applications is achieved. Experimental results show that the algorithm outperforms some existing algorithms in both classification and IQA applications.

Table 7 is constructed based on the above content. RR-IQA methods have progressively advanced from structural similarity and entropy-based analysis to perception-driven models and perceptual hashing frameworks, reflecting a consistent effort to enhance both efficiency and perceptual alignment. By integrating statistical modeling, human visual attention mechanisms, wavelet-domain analysis, and compressed sensing, these approaches achieve robust prediction of subjective quality with reduced reference requirements. Looking forward, the fusion of perceptual modeling and lightweight computational strategies is expected to further strengthen the scalability and applicability of RR-IQA in complex visual communication environments.

### 4.3. No-Reference Quality Assessment Method

NR-IQA is another important and challenging research branch in the field of IQA. Reference [86] systematically reviews NR-IQA methods, datasets, and challenges, proposes a classification framework based on distortion scenarios, analyzes the advantages and disadvantages of mainstream methods, discusses difficulties such as cross-device generalization and real-time performance, and looks forward to future research directions.

**Figure 7:**
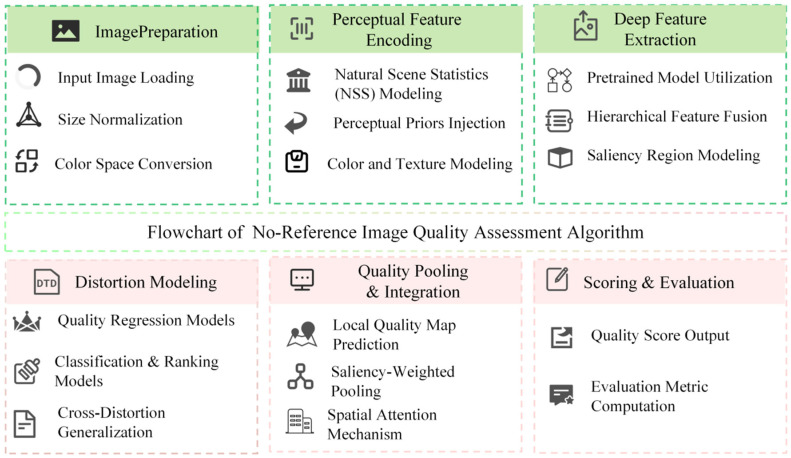
Flowchart of no-reference image quality assessment algorithm.

The NR-CIQA algorithm flow is shown in Figure 7.

#### 4.3.1. Methods Based on Feature Fusion

The method based on visual perception and feature fusion is an advanced method that combines computer vision and machine learning techniques. It aims to extract useful information from image or video data and improve the performance of the model by fusing multi-level and multi-modal features.

In 2019, Chen et al. [87] proposed an entropy-based no-reference image quality assessment (ENIQA) method. This method combines spatial and frequency domain features: the spatial domain characterizes the local structure and color correlation by calculating the two-dimensional entropy (TE) of the grayscale image and the mutual information (MI) between the RGB channels, and combines visual saliency to detect weighted key areas; the frequency domain uses Log–Gabor filtering to extract TE and MI of multi-directional and multi-band sub-band images to capture texture and contrast distortion. The two-stage framework of SVC and SVR is used to achieve distortion classification and quality regression. In 2022, Si et al. [88] proposed a no-reference stereo image quality assessment network StereoIF-Net based on binocular interaction and fusion mechanism. The method first extracts low-level visual features of the stereo image pair through two convolutional layers, and then designs four hierarchical binocular interaction modules (BIMs). The cross-convolution strategy models the interaction between left and right visual signals, simulating the binocular processing mechanism of the human cerebral cortex. Subsequently, a binocular fusion module (BFM) based on automatic learning weights is proposed to simulate the binocular fusion mechanism of the HVS high-level cortex and balance the weights of the left and right views. Finally, the final quality score of each image block is calculated through a local quality pooling strategy. Combining subjective scores with multi-dimensional features, StereoIF-Net shows excellent performance on the LIVE3D, IVC, and Waterloo-IVC SIQA databases, especially when dealing with symmetric and asymmetric distortions, which significantly improves the accuracy and generalization ability of the stereo image quality assessment. In 2023, Lan et al. [89] proposed an end-to-end blind image quality assessment (BIQA) method based on multi-level feature fusion. The method first uses a generative adversarial network (GAN) to convert the distorted image into a repaired image close to the reference image to supplement the information of the distorted image; then, the EfficientNet network is used to extract multi-level features of the repaired image and the distorted image in the Spatial-CIELAB color space, including details and semantic information of brightness, chroma, and hue. For the brightness component, multi-scale feature extraction is applied in both frequency and spatial domains to capture structural and contrast distortions, while, for chroma and hue, spatial-domain features are extracted directly to represent color distortions. Combining these multi-dimensional features, a bidirectional feature pyramid network (BiFPN) is used for feature fusion, the feature scores of each branch are calculated through the fully connected layer, and finally the image quality prediction score is averaged. In 2024, Lyu et al. [90] proposed an objective assessment index based on statistical color distribution (SCD). They constructed a semantically aware two-dimensional natural chromaticity distribution model through semantic segmentation, integrated neighboring area weighted smoothing, and probability density correction technology, and eliminated visual insensitivity and frequency anomaly interference. Yang et al. [91] proposed a restoration and quality feature learning NR-IQA method RQFL-IQA, which broke through the limitations of the existing separate optimization framework and for the first time incorporated distorted image restoration and quality feature learning into a unified model architecture. By simulating the hybrid loss function of the human brain repair mechanism, a closed loop of quality reconstruction relationships is constructed to achieve distortion repair and quality prediction simultaneously. By introducing multimodal label fusion and a reweighting mechanism, the method reduces interference from low-quality repair features and enhances feature mapping consistency with human perception. Zhao et al. [92] proposed a novel Multibranch Multilayer Feature Fusion Network (MFFNet) for NR-IQA. This method improves the fine-grained feature extraction capability through the multi-scale feature enhancement (MSFE) module of the main branch, uses the multi-layer feature fusion (MLFF) module to achieve cross-layer semantic information integration, and introduces the superpixel segmentation model of the sub-branch to capture local visual features. However, there are limitations such as high computational complexity, hyperparameter sensitivity, insufficient local feature expression, and strong dependence on the diversity of training data. In 2025, Sheng et al. [93] proposed a Latent Dirichlet Allocation Network for infrared image colorization, combining LDA color feature extraction with a multi-channel spatial attention mechanism to achieve accurate assessment of color fidelity and detail preservation.

Table 8 is constructed based on the above content. These methods have significantly improved the quality assessment performance in complex scenarios through strategies such as feature decoupling and multimodal fusion, especially in suppressing the interference of low-quality repair features and cross-modal feature mapping. However, existing methods still generally face the problem of feature space alignment and computational efficiency bottlenecks, which provides an important direction for improvement in subsequent research. Future research can focus on dynamic feature alignment networks, combined with cognitive-driven lightweight architectures, to break through feature compatibility constraints and achieve real-time quality assessment.

#### 4.3.2. Methods Based on Feature Extraction

Feature extraction is the process of extracting key information from an image that can characterize its content, aiming to reduce data complexity and retain features that are useful for the task. Traditional methods include edge detection, texture analysis, and color feature extraction, while deep learning methods automatically extract hierarchical features through convolutional neural networks.

Before 2019, Karen et al. [94] proposed a general NR-CIQA method. A color image chromaticity measurement model was defined, which is significantly consistent with human visual perception. The study proposed a grayscale image contrast measurement method based on NR contrast–Root Mean Enhancement (RME), further extending it to color images, and also proposed the NR color RME contrast measurement, exploring the three-dimensional contrast relationship of RGB color channels. Finally, based on the linear combination of color, clarity, and contrast, a color quality enhancement (CQE) measure was proposed.

In 2021, Tian et al. [95] proposed three image quality assessment metrics based on a comprehensive dataset (IQEMs): a color science-based model (CS), a neural network-based model (NN), and an image statistics-based model (IS). Subjective quality scores were collected to develop and validate these models through a large-scale psychophysical experiment involving 2266 color domain modified images. The neural network-based model (NN) achieved the highest prediction accuracy (R = 0.87) on both the training set and the test set, while the color science-based model (CS) performed relatively poorly. This study provides an effective method for color domain image quality assessment. In 2022, Zhang et al. [96] proposed a no-reference quality assessment method for color 3D point clouds and mesh models (NR-3D-IQA), which specifically evaluates the color and geometric structure distortion introduced by 3D models in operations such as simplification and compression. In terms of color distortion, the algorithm converts the RGB color space into the LAB color space and uses 3D natural scene statistics (3D-NSS) and entropy to quantify color deviation. In terms of geometric structure distortion, the algorithm extracts features such as curvature, anisotropy, linearity, flatness, sphericity of the point cloud, and curvature, dihedral angle, surface area, and angle of the mesh. Based on these features, a support vector regression (SVR) model is constructed, and the quality perception features and subjective scores are input into the model for training. Golestaneh et al. [97] proposed the Transformers, Relative Ranking, and Self-Consistency (TReS) method, which combines CNNs and Transformers to capture local and non-local features of the image, and introduces relative ranking and self-consistency mechanisms to improve assessment accuracy and robustness. Although it has excellent performance under various distortion types and has a strong generalization ability, this method is not targeted enough when dealing with specific distortion types, has high computational complexity, and has a certain dependence on the diversity of the data set. In 2024, Shi et al. [98] proposed a general NR-IQA method based on color moment and the Log–Gabor layer. The method first converts the input image to HSV color space to more accurately process the color information of the image. By extracting the color moment, the color gradient features in the image can be effectively captured. In terms of texture feature extraction, the Log–Gabor filter is used to divide the image into four layers to extract the texture information of each layer. Qiuhong et al. [99] proposed an NR-IQA method based on two-order color representation for color gamut mapping images. By fusing the zero-order and first-order color information, a regression model is constructed to quantify the texture and color naturalness loss. The introduction of color derivative dynamic features solves the common problem of incomplete feature representation in color gamut mapping quality assessment.

Table 9 is constructed based on the above content. These methods significantly improve the assessment accuracy in complex color gamut scenes through feature selection optimization and dynamic feature enhancement, but there are still technical bottlenecks in feature redundancy suppression and cross-device generalization. Future research can explore the adaptive feature compression framework to break through feature redundancy constraints and enhance cross-device generalization.

#### 4.3.3. Other Methods

There are also several other related research papers.

Before 2019, Maalouf et al. [100] proposed a multi-scale structure tensor clarity measurement method based on wavelet transform. This method effectively improves the sensitivity to image clarity by analyzing image structure information at multiple scales. Panetta et al. [101] proposed a new reference-free, parameter-free transform domain image quality measurement method—TDMEC. This method is designed for color images, does not rely on reference images, and does not require parameter adjustment during application.

In 2020, García-Lamont et al. [102] proposed a color image segmentation method that directly processes the RGB space without color space conversion. This method simulates the color perception mechanism of HVS by processing chrominance and intensity separately. Liu et al. [103] proposed an enhanced image NR-IQA model based on color space distribution: for a given enhanced image, GIST is first used to select a clear target image with similar scene, color, and quality to the assumed reference image; then, a reference image is constructed by color transfer between the input image and the target image; and finally the absolute color difference and feature similarity (FSIM) are used to measure the color and grayscale image quality, respectively. Experiments show that this method performs well in the enhanced quality assessment of X-ray, dusty, underwater, and low-light images, and the results are consistent with human subjective assessment. However, this method does not specify the specific matching criteria and accuracy verification method for GIST to select the target image. The impact of color transfer deviation is insufficiently analyzed, and the generalization ability of the model in more diverse enhanced scenarios is not explored. In 2021, Chen et al. [104] proposed a reference-free fuzzy color image quality assessment method based on dual maximum local information (DMLI). This method extracts local information from the image and combines the two features of maximum local difference and maximum local entropy to effectively evaluate the quality of the image. In 2023, Xu et al. [105] proposed a non-re-equalization assessment method based on the whole and high impact. Phase consistency-based structural features and opponent color space-based color features were extracted from the whole image and high-impact areas, respectively. Through a weighted strategy, the quality score of the high-impact area was used to moderately adjust the score of the whole image, thereby obtaining a more accurate IQA result. In 2024, Pérez-Delgado et al. [106] compared 10 color quantization (CQ) methods with 8 IQA indicators, and conducted experiments on the CQ100 dataset containing 100 RGB images, involving traditional indicators such as MSE and new indicators such as SSIM that consider HVS. The results show that the assessment results of traditional and new indicators are different, and it is recommended to combine multiple indicators to evaluate CQ methods. However, the consistency of the indicators with human perception was not verified through subjective experiments, nor was the optimal strategy for indicator combination established. Ibork et al. [107] proposed a reference-free three-dimensional color mesh visual quality assessment method Colored Mesh Visual Quality Assessment (CMVQA). This method combines geometric and color features with spatial domain features extracted from mesh projections. Miyata et al. [108] proposed a zero-shot interpretable no-reference image quality assessment method ZEN-IQA. Based on a pre-trained visual language model, this method evaluates image quality through antonym prompt pairs and triplets, provides an overall score and descriptive feature intermediate scores, and enhances interpretability. In 2025, Zhou et al. [109] proposed a NR-IQA method based on quality adversarial learning, which optimizes the quality prediction model by generating adversarial samples, emphasizing the balance between content fidelity and prediction accuracy. The design of the adversarial learning framework effectively improves the model’s perception of content fidelity. Ran et al. [110] studied the black-box adversarial attack method for the NR-IQA model and designed an attack algorithm based on a bidirectional loss function, which significantly reduced the performance of the mainstream NR-IQA model. A bidirectional loss function and an efficient black-box attack algorithm suitable for IQA tasks were proposed, revealing the model’s vulnerability to adversarial samples. Yang et al. [111] proposed a multi-scale dual-branch fusion NR-IQA method based on Vision Transformer (ViT). They used the self-attention mechanism of ViT and combined it with a multi-scale dual-branch fusion strategy to significantly improve the assessment accuracy and efficiency. However, the current method is limited by the size and diversity of the dataset.

Table 10 is constructed based on the above content. These methods range from multi-scale transformation [100], parameter-free measurement [101], RGB space processing [102], natural scene statistics [103], local information fusion [104,105], to three-dimensional grids [107] and zero-shot interpretable assessment [108], as well as adversarial learning and security research [109,110] and Transformer fusion [111]. Although each focuses on different feature extraction and assessment mechanisms, they all need to be optimized in terms of parameter adaptation, cross-channel complementarity, model robustness, and small sample generalization.

### 4.4. Specific Scene Color Image Quality Assessment

CIQA based on specific scenes or applications is optimized for specific scenes or specific needs. It includes but is not limited to the field of robotics, color gamut mapping, night image, and underwater image.

Gao et al. [112] proposed a new spatial domain color contrast enhancement algorithm based on an alpha-weighted quadratic filter. The characteristics of nonlinear filters are used to restore color information while enhancing contrast. The improved image contrast measurement index Global logAMEE is used to select the optimal parameters and verify the effectiveness of the algorithm. The proposed algorithm can effectively enhance contrast and color in the presence of noise and is suitable for real-time robotic applications.

Preiss et al. [113] focused on CIQA and proposed a color gamut mapping optimization algorithm with color image difference (CID) as the objective function, but the preliminary results have visual artifacts. The study obtained an improved color–image–difference (iCID) metric, which effectively avoids artifacts and improves image contrast, structure and color fidelity, while improving the prediction performance of visual data.

Wang et al. [114] focused on the night CIQA problem and proposed an NR-IQA method. This method deeply analyzes the characteristics of night images, divides the image locally according to the brightness level, and statistically analyzes the characteristics of local brightness information; it accurately measures the specific impact of weak lighting conditions on image color and structural information. Through the support vector regression technology, the extracted quality perception features are combined with subjective scores to construct a night IQA model. Song et al. [115] proposed a blind night-time image quality assessment method (BNTI) based on local and global feature analysis to address the quality assessment problems of night images caused by insufficient light, blurred structure, low contrast, and complex distortion. This method first extracts the local brightness features of the image through saliency analysis and exposure calculation, and quantifies the local contrast using the pixel value range; at the same time, the edge map entropy difference of the image and its enhanced version is calculated from a global perspective to characterize the structural distortion, and the color distortion is captured by the contrast energy in the opponent color space. Combining these multi-dimensional features, support vector regression (SVR) is used to build a quality assessment model to achieve accurate prediction of night image quality. Li et al. [116] proposed a perceptually calibrated synergy network (PCSNet) to jointly achieve night image quality prediction and enhancement. By sharing shallow networks to extract features and designing cross-sharing modules to optimize feature representation, the enhanced image information is used to calibrate quality assessment and promote multi-task collaboration.

Liu et al. [117] proposed a reference-free automatic colorization method based on generative adversarial networks (GAN) for the problem of automatic colorization of ethnic costume sketches. This method aims to solve the problem that ethnic costume sketches are rich in color and complex in pattern, and traditional colorization methods rely on professional experience and have low automation. A GAN model with a six-layer U-Net structure generator and a five-layer convolutional neural network discriminator is designed. By constructing an ethnic costume sketch database and using an edge detection algorithm to generate the sketch–color image pairs required for training, the model learns the color distribution law of ethnic costumes during training. The generator uses a U-Net structure to effectively retain image details. The discriminator extracts features through a convolutional layer and uses a fully connected layer to output the discriminant results. The smooth L1 loss function is combined to optimize the quality of the generated image.

Yang et al. [118] proposed a reference-free underwater color image quality assessment index (UCIQE) to address the challenges in the field of underwater CIQA. This index is based on the CIELab color space and achieves a comprehensive and objective assessment of underwater image quality by precisely extracting three key color image quality features: color contrast, saturation, and chroma. Panetta et al. [119] proposed novel reference-free Underwater Image Quality Measures (UIQM), which comprehensively consider the three major attribute indicators of underwater image color, clarity, and contrast. These indicators evaluate different dimensions of underwater image degradation, and their design is deeply inspired by the characteristics of HVS. Wang et al. [120] proposed a new underwater color image quality assessment index CCF, which is based on an in-depth analysis of the absorption and scattering characteristics of underwater imaging. CCF combines the color richness index, contrast index, and haze index, and quantifies the color loss caused by absorption, blur caused by forward scattering, and haze caused by backscattering by feature weighting. The weighting coefficients of these three key features are determined by the multivariate linear regression method. Chen et al. [121] proposed a UIQA method based on color space multi-feature fusion to address the problem of image quality degradation caused by the complexity of the underwater environment. The method first converts the underwater image from the RGB color space to the CIELab color space that is more consistent with human visual subjective perception, then extracts histogram features, morphological features, and moment statistical features from the brightness and color components and performs feature fusion. Using support vector regression technology, a prediction model between the fused features and the image quality score is constructed. Liu et al. [51] constructed a large-scale UIQA database UIQD, which contains 5369 real underwater images, covering a variety of scenes and typical quality degradation conditions, and becomes the UIQA standard database. A new baseline UIQA metric is proposed, which integrates channel and spatial attention mechanisms and transformer modules. The former captures image channels and local quality degradation, and the latter globally characterizes image quality. Multi-layer perception is used to fuse local and global features to obtain quality scores. In 2024, Dhivya et al. [122] proposed an underwater image quality assessment framework that integrates traditional models and deep learning. The framework constructs a multi-dimensional assessment system by synergistically optimizing the high-order representation of deep learning and the physical perception features of traditional models. Jiang et al. [123] proposed an explicit degradation-aware embedding network for UIQA. This method estimates the residual graph through the degradation information discovery subnetwork to characterize the local degradation situation, and embeds the degradation-aware features into the quality assessment network, thereby improving the accuracy of prediction.

Table 11 is constructed based on the above content. Scenario-specific CIQA methods cover areas such as robot vision contrast enhancement [112], color gamut mapping optimization [113], night-time low-light assessment [114,115,116], sketch colorization [117] and underwater quality assessment [51,118,119,120,121,122,123]. They improve the assessment accuracy by integrating scene features and multi-tasks, but they all face problems such as parameter sensitivity, high training cost, lack of real-time performance, and lack of cross-scenario generalization.

## 5. Discussion

### 5.1. Limitations of Existing CIQA Methods

In Section 4.1, Section 4.2 and Section 4.3, we systematically sorted out and studied the mainstream methods in the CIQA field. In order to further deepen the understanding of the methodology, this section will focus on the analysis of the limitations of the above method system, and will conduct an in-depth analysis of the shortcomings of various methods from the dimensions of machine learning methods, color visual quality and color characteristics, mathematical model methods, feature fusion methods, and feature extraction methods, as shown in Table 12.

(i) Limitations of machine learning-based methods

The CIQA methods based on machine learning have the following limitations: the twin network framework is prone to loss of global structural information due to the independence of dual-branch feature extraction, and multi-level pooling operations may cause detail attenuation; methods based on manual features are insufficient in generalization capabilities for complex distortion scenes, and modal conflicts are prone to occur when frequency domain and spatial domain features are fused; transfer learning methods are limited by the domain adaptability of pre-trained models, and the microscopic image enhancement process may introduce artifact interference; there is a semantic gap between physical features and depth representation in the hybrid architecture during collaborative optimization, and the scattering effect unique to underwater imaging is not explicitly modeled; etc.

(ii) Limitations of methods based on color visual quality and color characteristics

CIQA methods based on color visual quality and color characteristics have the following limitations: color similarity indicators are prone to failure under complex lighting conditions, and structural correlation modeling usually ignores high-order distortion coupling effects; manually designed saliency weighting strategies are not sensitive enough to dense texture areas and are difficult to adapt to multi-scale perceptual differences; registration methods that rely on reference images have reduced robustness in non-ideal scenes, and color card detection technologies have limited coverage of dynamic scenes or unconventional color gamuts; twin network architectures are prone to ignoring global contrast distribution due to local receptive field limitations, and statistical color models are prone to distribution estimation bias in edge fuzzy areas; etc.

(iii) Limitations of mathematical model-based methods

CIQA methods based on mathematical models have the following limitations: sparse representation methods have a limited ability to capture high-frequency texture details, and dictionary training relies on large-scale data sets; frequency domain analysis frameworks tend to ignore spatial local distortion distribution, and quaternion Fourier transform introduces the risk of phase information loss; gradient similarity metrics are less stable in noisy environments, and superpixel segmentation is prone to boundary quantization errors; saliency detection models are not sensitive enough to dense and repeated texture areas, and the sparse structural similarity assumption is prone to failure in complex distortion scenarios; etc.

(iv) Limitations of feature fusion-based methods

CIQA methods based on feature fusion have the following limitations: manually designed feature fusion strategies are difficult to adapt to multi-scale distortion coupling effects, and local–global feature collaborative modeling is prone to introduce modal conflicts; strong prior assumptions of domain-specific methods lead to reduced generalization ability, and Gaussian difference operators are sensitive to noise; multimodal label fusion depends on annotation quality, and mixed loss functions are prone to imbalance when optimizing restoration and quality prediction targets; attention mechanisms are prone to overfitting in dense texture areas, and masking effect models are not adaptable enough to complex lighting conditions; etc.

(v) Limitations of feature extraction-based methods

The CIQA method based on feature extraction has the following limitations: manually designed linear combination features tend to ignore the high-order distortion coupling effect, and the feature fusion strategy lacks an adaptive weight allocation mechanism; statistical features rely on ideal distribution assumptions and are prone to failure in complex distortion scenarios; color space conversion may introduce quantization errors, and the multi-scale analysis framework significantly increases the computational burden; frequency domain methods such as complementary color wavelet transform are susceptible to noise interference, and texture feature extraction is sensitive to rotation and scale changes; etc.

### 5.2. Future Research Directions

**Figure 8:**
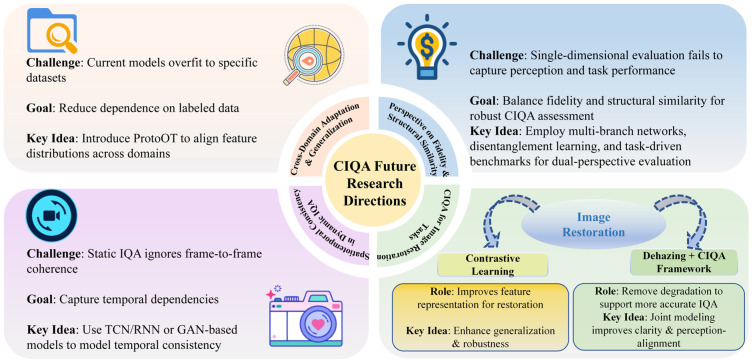
Outlook diagram.

As an important branch of image processing, computer vision, and artificial intelligence, CIQA has made some progress in many fields. However, as the demand for CIQA continues to expand, especially in applications with high precision and high timeliness requirements such as medicine, remote sensing, and virtual reality, existing research still faces many challenges. In this context, combining artificial intelligence, deep learning, and the latest technologies, we put forward some innovative ideas. Figure 8 shows the outlook diagram.

#### 5.2.1. Improvement of Cross-Domain Adaptation and Generalization Ability

Currently, most IQA methods perform well under specific datasets or distortion types, but their cross-domain generalization is weak. For novel image distortions, especially for cross-domain evaluation, existing methods typically require large amounts of labeled data for training and are susceptible to dataset bias. So, how can we design a universal and effective CIQA model that can adapt to diverse application domains across domains and reduce its reliance on specific datasets?

In this regard, ProtoOT (Prototype Optimal Transfer) [124] provides promising inspiration. Although ProtoOT was originally proposed for unsupervised cross-domain image retrieval, its core idea—constructing prototype representations in the source and target domains, aligning them through optimal transfer, and incorporating contrastive learning—addresses two key challenges facing CIQA: (i) the varying distribution of color-related features across domains; and (ii) the structural inconsistency of semantic content in the absence of distortion. By narrowing the domain gap at the prototype level, ProtoOT enables the model to transfer knowledge without requiring a large labeled target dataset.

Applying this paradigm to CIQA is both forward-thinking and practical. For example, prototypes can be constructed separately for fidelity-sensitive and structure-sensitive features. These prototypes can then be aligned across domains using optimal transfer, while contrastive learning can promote semantic consistency between distorted and no-reference samples. This adaptation is expected to significantly improve the robustness of CIQA when generalizing to new domains without requiring extensive relabeling. From this perspective, ProtoOT should be viewed as a methodological blueprint rather than a ready-made tool: it highlights that prototype-level alignment and contrastive optimization may be effective strategies for reducing bias in CIQA datasets, especially in high-risk domains such as remote sensing or medical imaging. This direction provides a concrete and innovative avenue for future CIQA research.

#### 5.2.2. A Dual Perspective on Fidelity and Structural Similarity

In CIQA research, image fidelity and structural similarity often influence subjective quality perception simultaneously, but they have different emphases: the former emphasizes accurate reproduction of pixel-level and color information, while the latter focuses more on preserving geometric relationships, edges, and textures. Given this, in color-sensitive or mission-critical applications, single-dimensional evaluation may not fully reflect subjective experience or downstream task performance. Therefore, it is necessary to more clearly distinguish and balance the two at the theoretical and experimental levels. To ensure the robustness of the demonstration, future work could construct test sets that distinguish between “fidelity distortion” and “structural distortion”, or group existing datasets by distortion type to test the sensitivity and generalization of different evaluation methods to these two types of distortion.

Methodologically, future research could advance the implementation of this dual perspective from several relatively feasible directions: First, multi-branch or two-stream networks can be used to learn fidelity-sensitive and structure-sensitive representations separately, followed by a learnable fusion strategy to adaptively weight them based on the task or scenario. Second, fidelity–structure disentanglement learning is expected to improve the discriminability and complementarity of the two components. Third, hybrid evaluation strategies and task-based benchmarks can be designed to clarify in which applications fidelity or structure should be emphasized, and weighting schemes for evaluation indicators can be formulated accordingly. Fourth, interpretability evaluation will facilitate diagnosis and improvement in engineering practice. Overall, prioritizing both fidelity and structural similarity as a research paradigm will help advance CIQA from general scoring to a more task-aware and interpretable evaluation system. However, the construction of relevant methods and benchmarks still needs to be verified through systematic experiments.

#### 5.2.3. Quality Assessment of Spatiotemporal Consistency

In the field of CIQA, the exploration of spatiotemporal consistency should not be regarded as an independent topic, but as a natural extension of static image quality assessment. Since a video sequence is essentially composed of consecutive static frames, the evaluation of video quality inevitably builds upon IQA. In the quality assessment of color videos and dynamic images, timing information plays a vital role. Distortions such as noise, compression artifacts, and color shifts first appear in individual frames, and CIQA provides the foundation for detecting these degradations. Therefore, VQA can be considered a higher-level task derived from CIQA, in which temporal information acts as a complementary dimension to frame-level quality.

Traditional CIQA methods mainly focus on the spatial characteristics of static images and often ignore the importance of the time dimension in dynamic scenes. However, the quality of video content depends not only on the spatial information of each frame, but also on the temporal information between frames. This interdependence highlights that CIQA and VQA are intrinsically linked, rather than completely separate fields. Future research can consider developing CIQA models that are extendable to dynamic scenarios based on the fusion of spatiotemporal features, using deep learning architectures such as temporal convolutional networks (TCN) or recurrent neural networks (RNN) to capture the temporal dependency between video frames. In addition, generative adversarial networks (GANs) can be adapted as a complement to frame-level CIQA in evaluating spatiotemporal consistency, providing guidance for video quality optimization and encoding. Anchoring VQA in CIQA methodologies is expected to achieve quality assessment of high-dynamic scene videos and, at the same time, highlight the indispensable role of static CIQA in advancing color video quality research.

#### 5.2.4. Image Restoration Problem

CIQA is a key means to measure the degree of quality restoration of the restored image, such as color and details. In the image restoration task, ensuring that the restored image meets the perceptual standards of HVS is the key to optimizing the restoration algorithm. CIQA provides a tool for quantitative assessment of the restoration results, so that the restoration algorithm can be continuously adjusted and optimized according to the quality assessment results. Therefore, CIQA provides feedback to the image restoration model to help optimize the image restoration effect. Conversely, image restoration technology also provides higher quality input for CIQA, improving the accuracy of quality assessment.

(i) Application of Contrastive Learning in Image Restoration

Contrastive learning, CIQA, and image restoration are closely related in the field of computer vision. As an unsupervised representation learning method, contrastive learning aims to obtain discriminative feature representations by maximizing the similarity of positive sample pairs and minimizing the similarity of negative sample pairs. In image restoration tasks such as denoising, super-resolution, and deblurring, contrastive learning is used to capture image degradation information and improve the generalization ability and robustness of the model. For example, researchers proposed an All-in-One [125] image restoration method that uses contrastive learning to achieve excellent performance in a variety of degradation scenarios. However, traditional IQA indicators often cannot accurately reflect the image quality perceived by the human eye. Therefore, CIQA, as an automatic assessment method that integrates the subjective scene imaging color and white balance of the camera, fully extracts the relevant features of color images and simulates the visual perception characteristics of the human eye to evaluate the image color.

Contrastive learning plays a role in improving model performance in image restoration, while CIQA provides an effective means to evaluate the perceived quality of restored images. The combination of the three is expected to promote the development of image processing technology and meet the needs of high-quality images in practical applications.

(ii) Application of Dehazing Model in Image Restoration

CIQA research can closely combine the dehazing model with image restoration technology to improve the accuracy and reliability of the assessment. As a kind of image restoration technology, the dehazing model aims to eliminate degradation factors such as haze in the image and restore the clarity and color information of the image. Traditional IQA methods mainly focus on basic visual features such as image clarity and contrast, but in complex environments these methods may not effectively reflect the true quality of the image. Therefore, if the dehazing model is combined with CIQA, it can provide a more accurate quality assessment on the basis of removing image degradation factors.

In addition, the integration of advanced deep learning technologies, such as GAN and adversarial training, can further enhance the robustness and adaptability of the assessment model, so that it can maintain high efficiency and stability in different application scenarios. In summary, the combination of dehazing models, CIQA, and image restoration technology not only provides new ideas for the further development of the dehazing task itself, but also opens up new research space in the field of CIQA.

## 6. Conclusions

This paper conducts a systematic study on CIQA and constructs a complete research framework of “application-data-algorithm-prospect”. At the application level, it sorts out image compression coding optimization, performance assessment of image processing and enhancement algorithms, and design of image quality optimization targets; for database construction, it introduces QACS, CCID2014, CEED2016, 4K Resolution Enhancement Artifact Database, and UIQD in detail; in terms of assessment indicators, it compares the applicable boundaries of PLCC, SROCC, MAE, RMSE, and Kendall rank correlation coefficient; it clarifies the complementarity of FR-CIQA and NR-CIQA methods—the former relies on reference images to achieve high-precision mapping, and the latter copes with complex scenes through feature fusion. The algorithm review goes deeper, from machine learning to visual perception models to mathematical modeling; it proposes potential directions for future research, mainly including the improvement of cross-domain adaptation and generalization capabilities, the advancement of fidelity–structure balancing, spatiotemporal consistency quality assessment, and the optimization of image restoration.

## Figures and Tables

**Table 1:** Overview of the dataset.

Dataset Name	Release Year	Number of Reference Images	Number of Distorted Images	Subjective Rating Range
LIVE	2006	29	779	DMOS [0, 100]
CSIQ	2010	30	866	DMOS [0, 1]
TID2013	2015	25	3000	MOS [0, 9]
QACS	2016	24	492	MOS [1, 10]
CCID2014	2016	15	655	MOS [1, 5]
CEED2016	2017	30	180	MOS [1, 5]
KonIQ-10k	2017	-	10,073	MOS [0, 5]
KADID-10k	2019	81	10,125	MOS [0, 5]
4K Resolution Enhancement Artifact Database	2022	24	1152	Subjective ratings provided by 20,000 observers

**Table 2:** Performance of PSNR on different databases.

Database	PLCC	SROCC	Kendall τ	RMSE	MAE
LIVE (DMOS 0–100)	0.8736	0.8781	0.6973	13.16	10.23
CSIQ (DMOS 0–1)	0.7921	0.8052	0.6080	0.1572	0.1254
TID2013 (MOS 0–9)	0.6213	0.6542	0.4852	1.213	0.9796

**Table 3:** Representative machine learning approaches in color image quality assessment.

Year	Author	Method/Model	Enabling Technologies	Major Contribution
pre-2019	Charrier et al. [57]	SVM-based FR-IQA	Multi-SVM classification; regression for quality scoring	Combined classification and regression to align better with human subjective perception
pre-2019	Ding et al. [19]	DISTS	CNN-based overcomplete representation; structural and texture similarity	Unified structure and texture similarity; robust to geometric distortions
2021	Ding et al. [59]	FR-IQA Optimization Study	Comparative evaluation of 11 FR-IQA models; DNN training for image enhancement	Evaluated perceptual performance of IQA models in low-level vision tasks
2022	Cao et al. [60]	Semi-supervised PU-Learning	Semi-supervised + PU learning; dual-branch network; spatial attention; local slice Wasserstein distance	Achieved high performance on benchmarks; solved misalignment in pseudo-MOS generation
2023	Lang et al. [61]	Conformer + Meta-learning	Conformer; twin network; meta-learning; global–local feature extraction	Enhanced generalization and competitive on standard datasets
2024	Reddy et al. [62]	ML-based IQA	Multi-feature descriptors; KNN matching; inlier ratio	Practical model using feature-based quality evaluation
2025	Lan et al. [63]	Hierarchical Degradation-Aware Net	Multi-level degradation simulation; similarity matrix; feature fusion + regression	High-precision quality scoring with improved adaptability to complex distortions

**Table 4:** Visual perception and color-based CIQA methods.

Year	Author	Method/Model	Enabling Technologies	Major Contribution
pre-2019	Thakur et al. [64]	Q_new_	HVS theory, brightness/structure/edge/color similarity	Overcame limitations of existing indices; suitable for diverse image processing tasks
pre-2019	Niu et al. [65]	Color Correction IQA Method	Color contrast similarity, color value difference	Improved accuracy of color consistency evaluation
2019	Kuo et al. [28]	Enhanced S-VIF	Integration of chroma channel into S-VIF	Improved performance by combining grayscale and color information
2020	Alsmadi et al. [66]	CBIR System	Color/shape/texture features, clustering, Canny, GA + SA	Achieved efficient and accurate image retrieval
2021	Cheon et al. [67]	Image Quality Transformer (IQT)	Transformer, CNN (Inception-ResNet-v2), multi-head attention	First Transformer in FR-IQA; modeled global distortion features
2022	Shi et al. [68]	FFS (Feature Fusion Similarity Index)	Three-feature fusion, symmetric calculation, bias pooling strategy	High consistency with subjective scores and efficient computation

**Table 5:** CIQA methods based on mathematical models.

Year	Author	Method/Model	Enabling Technologies	Major Contribution
pre-2019	Li et al. [69]	Sparse Representation + Residual	Overcomplete color dictionary, reconstruction residual, brightness similarity	Quantified structural, color, contrast distortions for accurate CIQA
pre-2019	Sun et al. [70]	SPSIM	Superpixel-based brightness/chromaticity/gradient similarity, texture complexity-based pooling	Achieved high subjective consistency by gradient-aware feature adjustment
2020	Shi et al. [71]	VCGS	Visual saliency, color appearance, gradient similarity, feature pooling strategy	Constructed a saliency-aware CIQA system with good consistency and moderate complexity
2022	Sun et al. [72]	Low-Light Enhancement + IQA	Multi-scale Retinex, ABC algorithm, histogram equalization, gamma correction	Enhanced low-light images with effective detail/noise balance via optimized fusion
2022	Varga et al. [73]	FR-IQA with GL Derivatives	Grünwald–Letnikov derivatives, image gradients, visual saliency weighting	Fused global/local changes for improved CVQ-consistent quality prediction
2024	Yang et al. [74]	IQA-SSSP	Sparse structural similarity, low-complexity computation, large-scale data processing	Integrated structure and perceptual similarity for scalable and efficient CIQA
2025	Bezerra et al. [75]	GL-Based IQA Method	Grünwald–Letnikov derivatives, image gradients, saliency-based perceptual weighting	Improved accuracy and consistency by combining global/local and perceptual features

**Table 6:** Supplementary CIQA-related research works.

Year	Author	Method/Model	Enabling Technologies	Major Contribution
pre-2019	Temel et al. [76]	Multi-Resolution IQA Method	LAB space, LoG, color similarity, retinal ganglion perception simulation	Simulated HVS perception by combining structural and color features
pre-2019	Liu et al. [77]	Color Distribution Model for Compression	Bright/median/dark channels, fractal dimension analysis	Used color distribution rules and fractal features to predict image quality
2019	Temel et al. [78]	SUMMER	Spectral error representation, multi-scale/ multi-channel features, color-aware spectral analysis	Enhanced color IQA by overcoming limitations of grayscale spectral analysis
2019	Athar et al. [79]	IQA Performance Assessment	Comparative benchmarking on 43 FR, 7 fusion FR, 14 NR methods across nine datasets	Provided comprehensive performance benchmarking of IQA methods
2023	Popovic et al. [80]	Saturation and Hue IQA Study	Subjective quality assessment, saturation/hue manipulation, correlation metrics	Built a dedicated dataset and validated subjective–objective correlation in scene perception
2025	Watanabe et al. [81]	FR-MLLM	Multimodal large language models, SVR fusion with traditional indicators	Proposed MLLM-based FR IQA for point clouds, enhancing accuracy through multimodal understanding

**Table 7:** Representative studies on reduced-reference IQA methods.

Year	Author	Method/Model	Enabling Technologies	Major Contribution
pre-2019	Soundararajan et al. [82]	SSIM-RR-IQA	SSIM theory, DNT-based statistical features	Simplified-reference IQA with strong correlation to SSIM and subjective scores
pre-2019	Rehman et al. [83]	RRED	Wavelet decomposition, entropy difference, error pooling	Entropy-based RR-IQA with reduced reference data and improved accuracy
pre-2019	Wang et al. [84]	RR-IQA for SCIs	Visual perception modeling, texture/edge metrics, attention mechanism	Efficient SCI quality prediction, effective for compression and visualization tasks
2022	Yu et al. [85]	CCWT-CS Perceptual Hashing	CCWT, compressed sensing, block-based feature extraction	Robust and compact perceptual hashing with superior IQA and classification performance

**Table 8:** Representative visual perception and feature fusion methods in NR-IQA.

Year	Author	Method/Model	Enabling Technologies	Major Contribution
2019	Chen et al. [87]	ENIQA	Spatial-frequency feature fusion, Log–Gabor filtering, TE and MI, visual saliency, SVC + SVR	Integrated spatial and frequency features using entropy and saliency; two-stage regression for NR-IQA
2022	Si et al. [88]	StereoIF-Net	Binocular interaction modules (BIM), binocular fusion (BFM), cross-convolution, local pooling	Modeled binocular vision mechanisms for stereo images; improved robustness to asymmetric distortions
2023	Lan et al. [89]	GAN + EfficientNet + BiFPN	GAN restoration, multi-level features in Spatial-CIELAB, BiFPN fusion, semantic and detail features	Combined GAN-repaired and distorted images for feature fusion; enhanced brightness, chroma, hue quality assessment
2024	Lyu et al. [90]	SCD-based Index	Statistical chromaticity distribution, semantic segmentation, smoothing, correction	Eliminated visual insensitivity and frequency anomaly interference
2024	Yang et al. [91]	RQFL-IQA	Joint restoration and quality learning, multimodal label fusion, perceptual reweighting	Unified distortion repair and quality assessment; simulated brain mechanism for enhanced perceptual consistency
2024	Zhao et al. [92]	MFFNet	MSFE module, MLFF module, superpixel-based sub-branch	Fused multi-layer and multi-branch features; improved local visual detail extraction despite computational cost
2025	Sheng et al. [93]	LDA Network	LDA-based color feature learning, multi-channel spatial attention	Applied LDA to infrared image colorization; enhanced fidelity and detail preservation in NR-IQA

**Table 9:** Representative feature extraction-based NR-IQA methods for color and 3D visual data.

Year	Author	Method/Model	Enabling Technologies	Major Contribution
pre-2019	Karen et al. [94]	CQE Measure	NR-RME contrast, color chromaticity, RGB 3D contrast, linear fusion	Proposed NR color contrast metric aligned with human perception; introduced CQE measure combining color, clarity, contrast
2021	Tian et al. [95]	IQEMs (CS, NN, IS)	Color science, image statistics, neural networks, large-scale subjective experiment	Developed three IQA metrics; NN model achieved highest accuracy (R = 0.87); validated on color-modified dataset
2022	Zhang et al. [96]	NR-3D-IQA	LAB color space, 3D-NSS, entropy, geometry features (e.g., curvature, angle), SVR	Assessed color and geometric distortions in 3D models using color and structural features with SVR training
2022	Golestaneh et al. [97]	TReS	CNN + Transformer fusion, relative ranking, self-consistency	Captured local/non-local features; improved generalization; limited specificity and higher computation cost
2024	Shi et al. [98]	HSV + Log–Gabor-based Model	HSV color moment, color gradient, Log–Gabor multi-layer texture extraction	Extracted effective color and texture features; improved NR-IQA accuracy for diverse distortions
2024	Qiuhong et al. [99]	Two-Order Color NR-IQA	Zero- and first-order color features, color derivative dynamics, regression model	Quantified texture and color naturalness loss in gamut mapping; addressed incomplete color representation

**Table 10:** Summary of selected research advances in color image quality assessment.

Year	Author	Method/Model	Enabling Technologies	Major Contribution
pre-2019	Maalouf et al. [100]	Multi-scale structure tensor clarity measurement	Wavelet transform and multi-scale image structure analysis	Improved sensitivity to image clarity by analyzing structure at multiple scales
pre-2019	Panetta et al. [101]	TDMEC (Transform Domain Image Quality Measurement)	Reference-free, parameter-free transform domain method	Reference-free IQA for color images without parameter tuning
2020	García-Lamont et al. [102]	Color image segmentation method	Direct RGB space processing, HVS-inspired chrominance, and intensity separation	Simulates HVS color perception without color space conversion
2020	Liu et al. [103]	Enhanced NR-IQA based on color space distribution	GIST for target image selection, color transfer, FSIM, and absolute color difference	Effective enhanced quality assessment for challenging images; noted limitations in target selection and generalization
2021	Chen et al. [104]	DMLI (Dual Maximum Local Information) NR-IQA method	Extraction of maximum local difference and local entropy features	Reference-free fuzzy color image quality evaluation combining two local information features
2023	Xu et al. [105]	Non-re-equalization IQA method	Phase-consistency structural features, weighted high-impact area scoring	Improved accuracy by weighting high-impact areas in image quality score
2024	Pérez-Delgado et al. [106]	Comparative evaluation of CQ methods	10 color quantization methods, 8 IQA indicators including MSE, SSIM	Recommended combining multiple indicators to evaluate CQ
2024	Ibork et al. [107]	CMVQA (Colored Mesh Visual Quality Assessment)	Combination of geometric, color, and spatial domain mesh features	Reference-free 3D color mesh visual quality assessment integrating multiple feature types
2024	Miyata et al. [108]	ZEN-IQA zero-shot interpretable NR-IQA	Pre-trained visual language model, antonym prompt pairs/triplets	Provides interpretable IQA with overall and intermediate descriptive scores
2025	Zhou et al. [109]	NR-IQA based on quality adversarial learning	Adversarial sample generation, content fidelity optimization	Improved model robustness and content fidelity perception via adversarial learning
2025	Ran et al. [110]	Black-box adversarial attack on NR-IQA models	Bidirectional loss function, black-box attack algorithm	Demonstrated vulnerability of NR-IQA models to adversarial attacks, proposing efficient attack method
2025	Yang et al. [111]	Multi-scale dual-branch fusion NR-IQA	Vision Transformer (ViT) with self-attention and multi-scale fusion	Enhanced accuracy and efficiency in NR-IQA, limited by dataset size and diversity

**Table 11:** Summary of specific scene color image quality assessment methods.

Year	Author	Method/Model	Enabling Technologies	Major Contribution
pre-2019	Gao et al. [112]	Spatial domain color contrast enhancement	Alpha-weighted quadratic nonlinear filter, Global logAMEE metric	Enhances contrast and color in noisy images; suitable for real-time robotics
pre-2019	Preiss et al. [113]	Color gamut mapping optimization	Color image difference (CID), improved iCID metric	Avoids visual artifacts; improves contrast, color fidelity, and prediction performance
2021	Wang et al. [114]	Night image NR-IQA method	Local brightness segmentation, support vector regression (SVR)	Models night image quality by analyzing local brightness impacts on color and structure
2021	Song et al. [115]	BNTI (Blind Night-Time Image QA)	Local/global brightness features, saliency, exposure, edge map entropy, SVR	Accurate night image quality prediction combining multidimensional features
2025	Li et al. [116]	PCSNet (Perceptually Calibrated Synergy Net)	Multi-task learning with shared shallow networks, cross-sharing modules	Joint night image quality prediction and enhancement through feature calibration and collaboration
2021	Liu et al. [117]	Reference-free automatic colorization	GAN with six-layer U-Net generator, five-layer CNN discriminator, edge detection	Automates ethnic costume sketch colorization with high detail retention
pre-2019	Yang et al. [118]	UCIQE (Underwater CIQA Index)	CIELab color space, extraction of color contrast, saturation, chroma features	Objective underwater image quality assessment addressing color degradation
pre-2019	Panetta et al. [119]	UIQM (Underwater Image Quality Measures)	Color, clarity, contrast metrics inspired by HVS	Comprehensive reference-free underwater image quality evaluation across major degradation dimensions
pre-2019	Wang et al. [120]	CCF (Underwater CIQA index)	Feature weighting via multivariate linear regression; color richness, contrast, haze indices	Quantifies underwater image degradation caused by absorption, scattering, and haze
2023	Chen et al. [121]	UIQA based on multi-feature fusion	CIELab conversion; histogram, morphology, moment features; SVR	Addresses underwater image complexity via multi-feature fusion and quality score prediction
2024	Dhivya et al. [122]	Hybrid Underwater IQA	Traditional model fusion; deep learning high-order feature representation	Built multi-dimensional underwater image quality assessment framework
2024	Liu et al. [51]	Baseline UIQA metric and UIQD database	Channel/spatial attention, transformer modules, multi-layer perceptron fusion	Large-scale underwater image database; integrated local–global feature-based quality assessment
2025	Jiang et al. [123]	Degradation-aware embedding network for UIQA	Residual graph estimation, degradation information subnetwork, feature embedding	Improves underwater image quality prediction by modeling local degradation explicitly

**Table 12:** Analysis of image quality assessment methods.

Method Type	Mechanism	Advantages	Limitations
Machine Learning	Uses CNNs, meta-learning, transfer learning with regression/classification	Handles complex distortions; supports weak supervision; enhances generalization	Requires large data; twin networks lose global info; semantic gaps exist
Color Visual Quality and Color	Inspired by the human visual system (HVS); extracts features such as color difference, structural similarity, and saliency	Good perceptual alignment; handles structure and color variation	Fails under complex lighting; ignores high-order distortions; poor reference registration
Mathematical Models	Uses sparse coding, Fourier analysis, gradient similarity, statistical modeling	Interpretable; suitable for low-level structure modeling	Weak for fine texture; frequency models lack spatial sensitivity
Feature Fusion	Fuses spatial–frequency, color–structure, subjective–physical features	Combines diverse cues; improves robustness and cross-domain performance	Manual fusion design; modal conflict risk; loss balancing difficult
Feature Extraction	Extracts texture, color, statistical features for quality modeling	Simple and interpretable; good for lightweight applications	Lacks adaptivity; weak for high-order distortions; may incur high cost

## Data Availability

No new data were created or analyzed in this study.
